# 942. Pulmonary Infections in Intestinal Transplant Recipients with Preexisting Pulmonary Nodules

**DOI:** 10.1093/ofid/ofab466.1137

**Published:** 2021-12-04

**Authors:** Jorge Cardenas, Yoichiro Natori, Shweta Anjan, Rodrigo Vianna, Jennifer Garcia, Jacques Simkins

**Affiliations:** 1 University of Miami, Miami, FL; 2 Jackson Memorial Hospital/Miami Transplant Institute, University of Miami Miller School of Medicine, Miami, FL; 3 University of Miami/Jackson Memorial Hospital, Miami, FL; 4 Jackson Memorial Hospital/ Miami Transplant Institute, Miami, FL

## Abstract

**Background:**

Pulmonary nodules in asymptomatic patients could represent latent pulmonary infections. Intestinal transplant (ITx) recipients with preexisting pulmonary nodules might be at higher risk for pulmonary infections. However, data is lacking.

**Methods:**

This retrospective study included adult patients that underwent intestinal transplantation (ITx) from 5/2016 to 5/2020. Chest computed tomography (CT) scans performed within 12 months prior of ITx were obtained to evaluate for preexisting pulmonary nodules. Screening for endemic mycoses, *Aspergillus*, *Cryptococcus* and latent tuberculosis infection (LTBI) performed within 12 months prior ITx was obtained. We assessed for worsening pulmonary nodules, and fungal and mycobacterial infections during the 1^st^ year post-transplant. Survival at one year post-transplant was also assessed.

**Results:**

Forty-three patients underwent ITx. Twenty-three (53%) were Female. Median age was 46 years (range: 18-67). Chest CT scans were performed in 36(84%) patients prior to ITx. Preexisting pulmonary nodules were found in 30 (83%) of the patients. All were asymptomatic. Nodules were not calcified in 10 (33%) patients, calcified in 4 (13%), some calcified and some not calcified in 4 (13%) and unclear in 12 (40%). All the patients screened negative for fungi [*Coccidioides* antibody (Ab) was done in 15 (50%) patients, *Blastomyces* Ab and Histoplasma Ab in 7 (23%) each, *Histoplasma* urine antigen (Ag) and *Aspergillus* serum galactomannan in 3 (10%) each, and *Cryptococcus* serum Ag in 10 (33%) patients]. QuantiFERON-TB (QFT) was negative in 35 (81%) patients, positive in 2 (5%) and indeterminate in 6 patients (14%). QFT-Gold In-Tube was replaced to QFT-Plus in 3/2019. Post-transplant worsening of pulmonary nodules was noted in 12 (40%) patients and bronchoscopy was performed in six of them. Note that only 1 (3%) of the patients that had pre-existing pulmonary nodules developed a pulmonary infection (invasive pulmonary aspergillosis diagnosed 33 days after ITx). Our cohort survival at one year post-transplant was 79%.

**Conclusion:**

Preexisting pulmonary nodules was common in our ITx cohort. However, only one case of pulmonary infection was noted among those who had preexisting pulmonary nodules. Clinical monitoring is essential.

**Disclosures:**

**All Authors**: No reported disclosures

